# Electrochromic properties of WO_3_ thin films: The role of film thickness

**DOI:** 10.1002/ansa.202000072

**Published:** 2020-07-18

**Authors:** Yingpeng Zhen, Bjørn Petter Jelle, Tao Gao

**Affiliations:** ^1^ Department of Civil and Environmental Engineering Norwegian University of Science and Technology (NTNU) Trondheim Norway; ^2^ Department of Materials and Structures SINTEF Community Trondheim Norway

**Keywords:** electrochromic windows, nanofilm thickness, smart windows, thin nanofilm, tungsten oxide

## Abstract

Tungsten oxide (WO_3_) thin films with various thicknesses of approximately 36, 72, 108, and 180 nm were prepared using radio frequency sputtering method. Film thickness can be controlled at nanoscale. In addition, X‐ray diffraction, scanning electron microscopy, and Fourier transform infrared spectroscopy were utilized for investigating morphologies and microstructures of as‐prepared WO_3_ thin films. Moreover, optical properties of the WO_3_ nanofilms were characterized using ultraviolet‐visible‐near infrared spectroscopy. Transmittance of WO_3_ films changed during the electrochemical cycles. WO_3_ films with various thicknesses give various transmittance modulation between colored and bleached states. WO_3_ films with a thickness of approximately 108 nm had the largest transmittance modulation among various film thicknesses, about 66% measured at 550 nm. Results showed that the value of transmittance of colored samples decreased with increasing film thickness. However, transmittance of bleached samples was not influenced significantly by their thickness.

## 1 INTRODUCTION

Energy consumption and greenhouse gas emission of buildings account for a large part of total energy consumption and CO_2_ emissions in both developed and developing countries in the world. A building may lose about 30% of its heat or air conditioning energy through its windows, because windows have usually poor thermal insulation properties and high radiation transmittance. Windows with low *U*‐value (*U*‐value of window can be utilized to measure heat transfer rate through window; normally, window with a lower *U*‐value will have a lower amount of heat loss, and will also have a better thermal insulation property) have been widely used in buildings to prevent heat loss by heat transfer (thermal conduction, thermal convection, and radiation). Chromogenic smart windows have been used for regulating solar radiation transmitted through the windows into buildings. Therefore, energy consumption of buildings can be reduced. However, nowadays, chromogenic smart windows are not widely utilized in normal buildings.

Chromogenic smart windows can adjust solar radiation transmitted through windows by regulating transmittance value. Glass with functional film of chromogenic smart windows can change windows transmittance value if functional film materials of glass are sensitive to voltage, solar radiation, heat, and so on. Different chromogenic smart windows are made of different chromogenic materials. Based on various chromogenic materials, there are photochromic smart windows, thermochromic smart windows, gasochromic smart windows, and electrochromic smart windows.

Electrochromic material is a type of chromogenic material. Electrochromic material is sensitive to an external applied voltage. The most popular electrochromic material is transition metal oxide, which may be divided into two types: (a) anodic coloration materials and (b) cathodic coloration materials. Anodic coloration materials include NiO,^1^ Co_2_O_3_,^2^ Ir(OH)_3_,^3^ V_2_O_5_,^4^ and so forth. The cathodic coloration materials include tungsten oxide (WO_3_),^5^ MoO_3_,^5^, ^6^ TiO_2_,^7^ and so forth. Many different electrochromic polymers have also been investigated.^8^, ^9^, ^10^

WO_3_ is one of the most studied electrochromic materials for electrochromic smart window applications. There are many kinds of methods utilized for WO_3_ film preparation, such as evaporation,^11^ sputtering,^12^ electrodeposition,^13^ chemical vapor deposition,^14^ and sol‐gel deposition.^15^

Electrochromic materials and devices^16^, ^17^, ^18^, ^19^, ^20^, ^21^, ^22^ were investigated in our previous work. WO_3_ thin films were prepared using a radio frequency (RF) sputtering technology, which enables to control the film thickness accurately at nanometer scale. WO_3_ thin films with various thicknesses were prepared. The role of the film thickness on structural and optical properties of WO_3_ films was investigated. Microstructure of as‐prepared WO_3_ thin films was characterized using X‐ray diffraction (XRD), scanning electron microscope (SEM), and Fourier transform infrared (FTIR) spectroscopy. Electrochemical properties of as‐prepared WO_3_ films were characterized using cyclic voltammetry (CV). Ultraviolet‐visible‐near infrared (UV‐Vis‐NIR) spectroscopy was utilized to analyze optical properties of WO_3_ films.

## 2 EXPERIMENTAL SECTION

### 2.1 Materials

WO_3_ sputtering target (diameter = 5.08 cm; thickness = 0.32 cm) was purchased from AJA International Inc., USA. Substrates for growth of WO_3_ thin films were ITO (tin doped indium oxide, In_2_O_3_(Sn)) glass slides, which have been purchased from Sigma‐Aldrich (Transmittance: approximately 86% at 550 nm; Surface resistivity: about 70‐100 Ω/square; Size: about 25 mm × 25 mm × 1.1 mm). The ITO glass slides were cut to small pieces with sizes of about 9.2 mm × 25 mm × 1.1 mm in this work. Reagent grade sulfuric acid (H_2_SO_4_, 96 wt%) and potassium chloride (KCl, 99%) were also purchased from Sigma‐Aldrich. Distilled water from our laboratory was utilized in this work.

### 2.2 Preparation of WO_3_ films

First, ITO glass slides were washed with ethanol under ultrasonic irradiation. And then ITO glass slides were washed with distilled water. In the end, ITO glass slides were dried using nitrogen gas. Preparation process of WO_3_ films was similar to our previous work.^23^ In brief, WO_3_ films were prepared by RF magnetron sputtering via an AJA sputter and evaporator (model: Custom ATC‐2200V, AJA International Inc. USA). RF power was set to 120 W and the ramp up time was 210 s. Sputtering rate has been checked as 0.05 nm/s before the sample sputtering process. ITO glass substrates were loaded into sample holder. Argon (Ar) flow was 67 sccm (standard cubic centimeter per minute). Plasma strike pressure was 30 mTorr (approximately 4.0 Pa) Ar pressure. Strike power is 50 W. After plasma was strike on, Ar pressure was changed and maintained at 3 mTorr (approximately 0.4 Pa) during plasma processes. After RF power reached set value, 1 min was spent to wait for the stability of plasma. Subsequently, the shutter that covered the target is opened during sputtering process. And then the shutter was closed as long as the desired film thickness was reached, for example, 720 s for film with thickness as 36 nm. In the end, after the power was decreased to 50 W, the plasma was turned off.

Heat treatment was also performed to the sputtered films in air at 400°C for 5 h. By this way, as‐prepared samples were obtained in this work for further investigation.

### 2.3 Characterization

XRD patterns of as‐prepared samples were collected on a D8 A25 DaVinci X‐ray diffractometer (Bruker, Germany) with Cu‐Kα radiation. XRD data were collected in the range of 15°‐75° with a step size of 0.013°. The surface topography of samples was investigated using an Apreo SEM from Thermo Fisher Scientific (FEI, USA). Attenuated total reflectance (ATR) FTIR spectra were recorded on a Nicolet 8700 FTIR Spectrometer (Thermo Scientific, USA). A horizontal single‐bounce ATR diamond accessory was utilized for FTIR characterization. The spectra were recorded in wavenumbers ranging from 400 to 4000 cm^−1^ at a spectral resolution of 2 cm^− 1^.

CV measurements were conducted using a potentiostatic procedure at a scan rate of 20 mV/s in the potential range from –0.5 to 1.0 V versus Ag/AgCl on an AutoLab PGSTAT302N electrochemical workstation (Metrohm Autolab B.V., Netherlands). A standard three‐electrode electrochemical cell was assembled, where a WO_3_ film, a Pt film, and a reference electrode (Ag/AgCl, 3 M KCl) were used as working electrode, counter electrode, and reference electrode, respectively. The electrolyte was 0.5 M H_2_SO_4_ aqueous solution. Experiments were performed under atmospheric environment.

Transmittance of samples was measured in the wavelength ranging from 280 to 1300 nm using a UV‐Vis‐NIR spectrophotometer (PerkinElmer 1050 WB, USA). Samples were measured in situ when they were utilized as working electrodes in an electrochemical system. Because H_2_SO_4_ solution electrolyte had a strong absorption to light with wavelength above 1350 nm. Transmittance spectra were recorded from 280 to 1300 nm. A constant potential of –0.5 and +1.0 V (vs Ag/AgCl, 3 M KCl) was used to color and bleach samples, respectively.

## 3 RESULTS AND DISCUSSIONS

### 3.1 X‐ray diffraction

**FIGURE 1:**
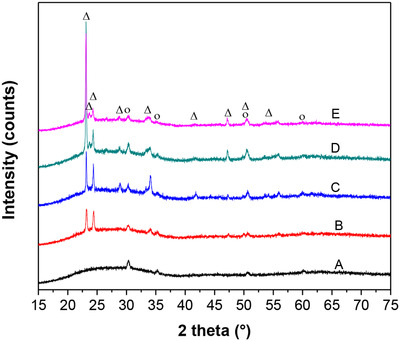
XRD results of as‐prepared WO_3_ nanofilm samples: (A) ITO glass, (B) 36‐nm WO_3_ nanofilm, (C) 72‐nm WO_3_ nanofilm, (D) 108‐nm WO_3_ nanofilm, and (E) 180‐nm WO_3_ nanofilm. Open circles (O) denote the reflections of ITO (Powder Diffraction File [PDF] 04‐019‐3926). Open triangles (∆) denote the reflections of monoclinic WO_3_ (PDF 00‐043‐1035)

Figure 1 depicts XRD patterns of WO_3_ films with four various thicknesses, namely, 36, 72, 108, and 180 nm. XRD peaks of ITO substrates (Figure 1A) matched well with the work of Kim et al.^24^ All WO_3_ films showed similar diffraction peaks (Figure 1B‐E). The peaks at 23.1°, 23.6°, and 24.4° can be assigned to (002), (020), and (200) reflections of the monoclinic WO_3_ (Powder Diffraction File 00‐043‐1035), respectively. XRD results showed that as‐prepared WO_3_ films with various thickness of 36, 72, 108, and 180 nm had the same monoclinic crystal structure.

### 3.2 Fourier transform infrared spectroscopy

**FIGURE 2:**
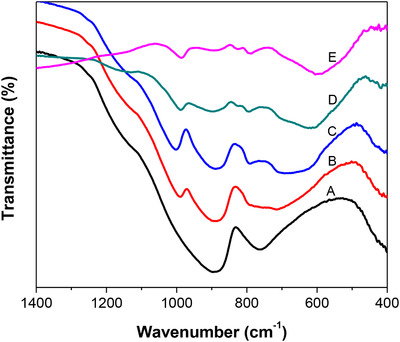
FTIR spectra of as‐prepared WO_3_ nanofilm samples: (A) ITO glass, (B) 36‐nm WO_3_ nanofilm, (C) 72‐nm WO_3_ nanofilm, (D) 108‐nm WO_3_ nanofilm, and (E) 180‐nm WO_3_ nanofilm

Chemical structure of the obtained films was also characterized using FTIR (as shown in Figure 2). FTIR results showed that there were absorption peaks at approximately 900 and 760 cm^–1^ for ITO glass, which was in agreement with the work of Meng and Santos.^25^ The absorption peak at about 980 cm^–1^ is related to W = O stretching vibrations, whereas the absorption peak at about 585 cm^–1^ is corresponding to O‐W‐O stretching vibrations from the crystalline WO_3_.^18^, ^26^ FTIR results showed that as‐prepared WO_3_ films with various thickness of 36, 72, 108, and 180 nm had similar crystalline structure. The FTIR result was in agreement with XRD result.

### 3.3 Scanning electron microscope

**FIGURE 3:**
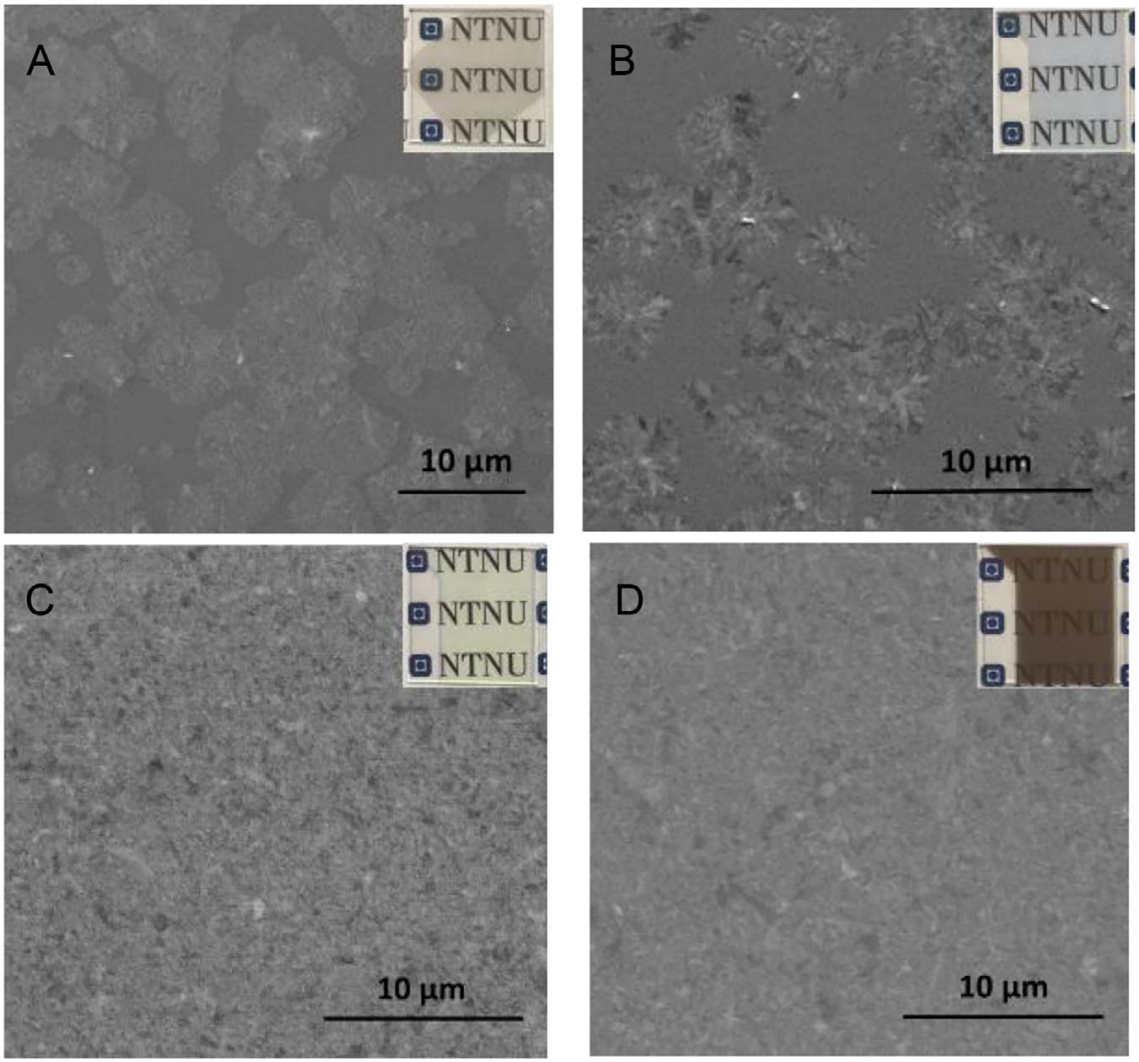
Photos of glass with as‐prepared WO_3_ films and SEM images of as‐prepared WO_3_ films with various thicknesses: (A) 36 nm, (B) 72 nm, (C) 108 nm, and (D) 180 nm WO_3_ film

Morphology of WO_3_ films was investigated using SEM. As shown in Figure 3, crystals were formed in all the as‐prepared WO_3_ films. These films showed different colors, including light gray, light blue, lemon green, and brown. The crystals did not cover the whole substrate completely when the WO_3_ film thickness was 36 and 72 nm (as shown in Figures 3A and 3B). However, as was shown in samples of WO_3_ film with thickness of 108 and 180 nm, with the increase of film thickness, crystals covered almost the whole substrate (Figures 3C and 3D).

### 3.4 Cyclic voltammetry

**FIGURE 4:**
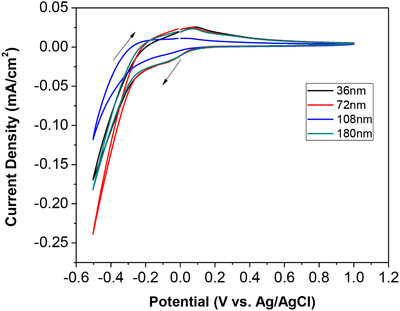
CV curves of WO_3_ thin films with various film thicknesses of 36, 72, 108, and 180 nm at a scanning rate of 20 mV/s

Electrochemical properties of as‐prepared WO_3_ films were investigated by CV method, which was carried out between –0.5 and 1.0 V versus Ag/AgCl (3 M KCl) at a scan rate of 20 mV/s. Figure 4 showed the CV curves experimental results. WO_3_ thin films with various thicknesses exhibited similar CV curves. A cathodic current peak emerged at around –0.1 V and an anodic current peak appeared at around 0.1 V. The two peaks were accompanied by the coloration (from colorless to blue, ie, W^6+^ → W^5+^) and bleaching (from blue to colorless, ie, W^5+^ → W^6+^) of the WO_3_ films, respectively. The redox peaks were not influenced by the film thickness of WO_3._

### 3.5 UV‐Vis‐NIR spectroscopy

**FIGURE 5:**
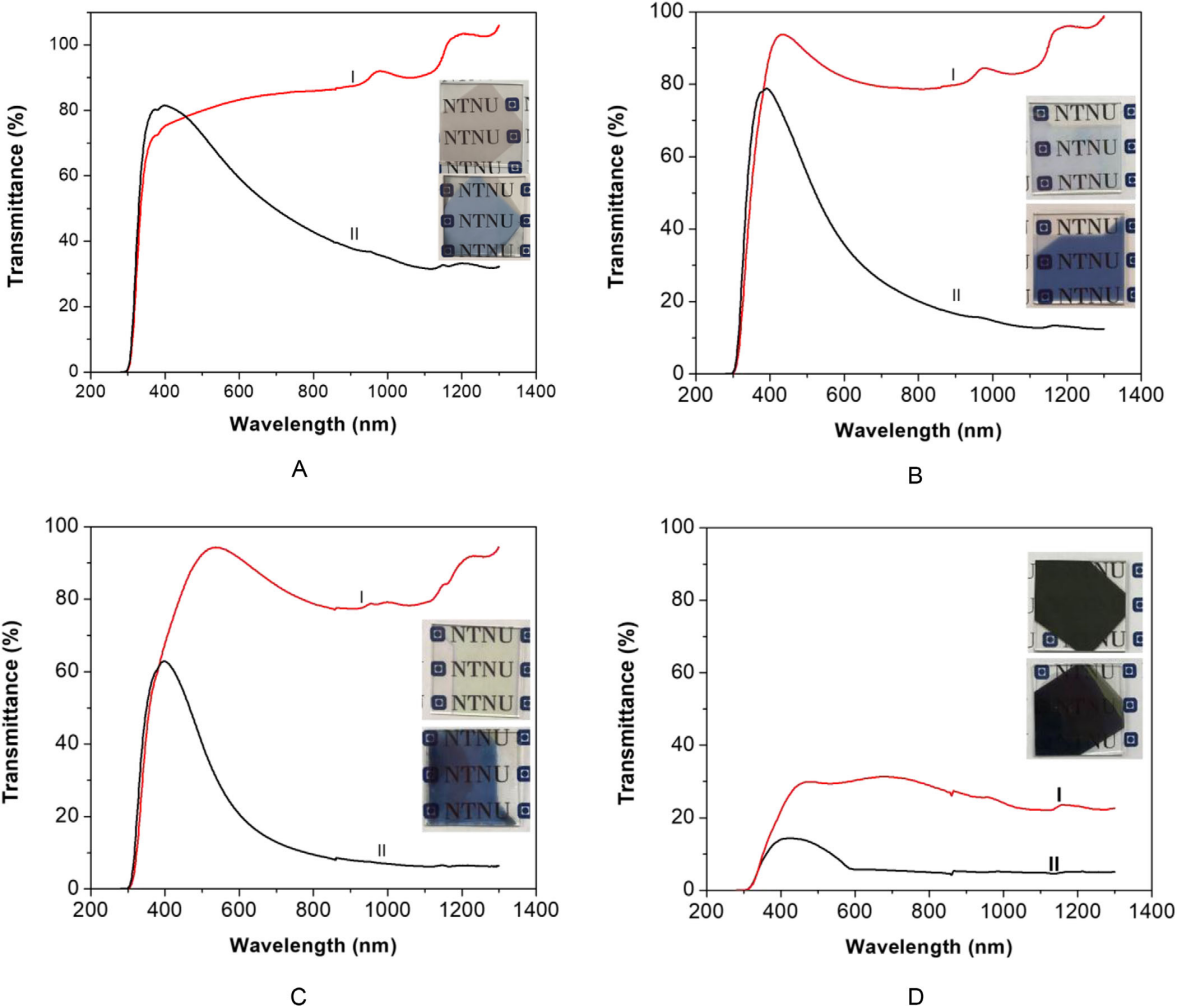
Transmittance spectra of WO_3_ thin‐film samples with various film thicknesses: (A) 36 nm, (B) 72 nm, (C) 108 nm, and (D) 180 nm in the bleached state (I) and colored state (II). Inset shows the corresponding photos of as‐prepared WO_3_ films at bleached (up) and colored (down) state (measured in 0.5 M H_2_SO_4_)

UV‐Vis‐NIR spectroscopy was utilized to characterize WO_3_ films. The colored samples were obtained by applying an external potential voltage of –0.5 V to as‐prepared samples for 5 h, whereas the transmittance of bleached samples were obtained from as‐prepared samples before any external potential voltage was applied. Bleached experiments were performed by applying an external potential voltage of +1.0 V to as‐prepared samples for 5 h. However, it was found that the transmittance of bleached samples after an external potential voltage of +1.0 V was applied for 5 h was almost the same as they were before an external potential voltage of +1.0 V was applied. Transmittance spectra of WO_3_ films were shown in Figure 5. The samples were measured in a self‐made cuvette filled with 0.5 M H_2_SO_4_. As shown in Figure 5A, at a wavelength of *λ* = 550 nm, the transmittance value (*T*) of the 36 nm sample was about 66% at the colored state and 82% at the bleached state. This resulted in a transmittance modulation (*∆T*) of about 16% at 550 nm (∆*T*_550 nm_).

The transmittance spectra of the colored and bleached samples with thicknesses of 72, 108, and 180 nm were shown in Figures 5B, 5C, and 5D, respectively. *T* of the samples in the bleached state and colored state at a wavelength of *λ* = 550 nm were obtained and given in Table 1. ∆*T*_550 nm_ of the samples with thickness of 36, 72, 108, and 180 nm were 16%, 41%, 66%, and 21%, respectively. From these results, it can be seen that the as‐prepared WO_3_ film with thickness of 108 nm had the largest *∆T* of about 66% at 550 nm.

The largest*T* of bleached samples was obtained from WO_3_ film with thickness of 108 nm. It is shown that the WO_3_ coating thickness increase did not always make*T* decrease with the increasing of nanofilm thickness from 36 nm to 180 nm.*T*_550 nm_ of bleached films decreased with nanofilm thickness of 108 nm (highest transmittance), 72 nm, 36 nm and 180 nm (lowest transmittance).

**FIGURE 6:**
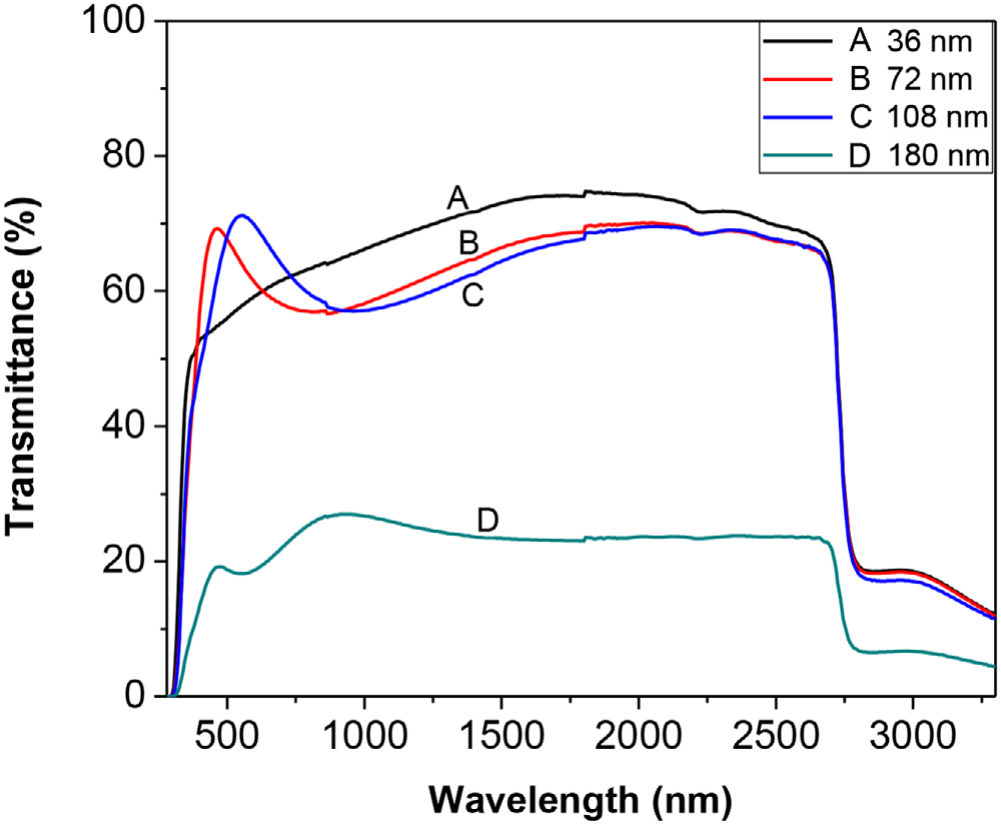
Transmittance spectra of WO_3_ thin film with various film thicknesses: (A) 36 nm, (B) 72 nm, (C) 108 nm, and (D) 180 nm at the bleached state (measured in air)

To further prove results above, transmittance of bleached samples (as prepared WO_3_ films without further treatment) were measured in air. The transmittance curves of the bleached samples were shown in figure 6.

**TABLE 1:** Transmittance of WO_3_ films at colored and bleached states at a wavelength of *λ* = 550 nm (measured in 0.5 M H_2_SO_4_ aqueous electrolyte solution)

WO_3_ film thickness (nm)	*T*_550 nm_ bleached state (%)	*T*_550 nm_ colored state (%)	*∆T*_550 nm_ between bleached and colored state (%)
36	82	66	16
72	85	44	41
108	94	28	66
180	29	8	21

Figure 6 depicts the transmittance spectra of bleached WO_3_ films in the wavelength ranging from 280 to 3300 nm, which were measured in a standard optical glass cuvette with no liquid filled. It was shown that the transmittance order was different at different range of wavelength. Transmittance of bleached films decreased by the increase of thickness in the wavelength ranging from 935 to 3300 nm. However, in the wavelength ranging from 390 to 495 nm, the film with thickness of 72 nm had the highest transmittance among all the films utilized in this work. At wavelength ranging from 495 to 740 nm, the film with thickness of 108 nm had the highest transmittance compared to the other films. From Figure 6, the order of transmittance values of various bleached films at wavelength of 550 nm was 108‐nm film (highest transmittance), 72‐nm film, 36‐nm film, and 180‐nm film (lowest transmittance). These results were in agreement with results showed in Table 1.

## 4 CONCLUSIONS

In this work, WO_3_ thin films with various thicknesses of approximately 36, 72, 108, and 180 nm were prepared. Morphologies and microstructures of as‐prepared WO_3_ thin films were characterized using XRD, SEM, and FTIR. In addition, UV‐Vis‐NIR spectroscopy was utilized for transmittance investigations of WO_3_ films. The bleached WO_3_ films showed color as light gray, light blue, lemon green, and brown, whereas the colored films showed color as blue with different transmittance levels. WO_3_ films with various thicknesses lead to various transmittance values at the colored and bleached state. Among WO_3_ film samples with thickness of 36, 72, 108, and 180 nm, the largest transmittance modulation ∆*T*_550 nm_ was obtained from sample with thickness of 108 nm, which was approximately 66% when measured in 0.5 M H_2_SO_4_. It showed that the value of transmittance of colored samples decreased with increasing of their thickness, whereas the transmittance of bleached samples was not affected by their thicknesses. This work will benefit electrochromic smart window development.

## CONFLICT OF INTEREST

The authors declare no conflict to declare.
